# Study of the Mechanism of Action of Guanxin Shutong Capsules in the Treatment of Coronary Heart Disease Based on Metabolomics

**DOI:** 10.3389/fphar.2021.650438

**Published:** 2021-03-25

**Authors:** Dan Wang, Chang Shi, Zhen-Hua Ge, Yu-Xi Wei, Tian-Tian Liu, Yue Wang, Xin-Feng Zhou, Zi-Jun Yang, Wei-Ting Wang, Yan-Wen Zhang, Xue-Hui Zhu, Jun Zhang, Ying Li, Min Gong, Xiao-Hui Wu, Hong-Quan Duan

**Affiliations:** ^1^ College of Pharmacy, Tianjin Medical University, Tianjin, China; ^2^ Department of Pharmacy, Tianjin Huanhu Hospital, Tianjin, China; ^3^ Department of Pharmacy, Tianjin Hospital, Tianjin, China; ^4^ Department of Anesthesiology, Pharmacology and Therapeutics, the University of British Columbia, Vancouver, BC, Canada; ^5^ School of Medical Humanities, Tianjin Medical University, Tianjin, China; ^6^ Tianjin Institute of Pharmaceutical Research, Tianjin, China; ^7^ Tianjin Neurological Institute, Tianjin Medical University, Tianjin, China

**Keywords:** metabolomics, Coronary heart disease, traditional Chinese medicine, Guan-Xin-Shu-Tong capsule, active ingredients

## Abstract

**Background**: Guan-Xin-Shu-Tong capsule (GXSTC) is a traditional Chinese medicine (TCM) that has been used to treat coronary heart disease (CHD) for many years in China. However, the holistic mechanism of GXSTC against CHD is still unclear. Therefore, the purpose of this paper was to systematically explore the mechanism of action GXSTC in the treatment of CHD rats using a metabolomics strategy.

**Methods**: A CHD model was induced by ligation of the left anterior descending coronary artery (LAD). In each group, echocardiography was performed; the contents of creatine kinase (CK), lactate dehydrogenase (LDH) and aspartate transaminase (AST) in serum were determined; and the myocardial infarct size was measured. The metabolites in plasma were analyzed by UHPLC-MS/MS-based untargeted metabolomics. Then, multivariate statistical analysis was performed to screen potential biomarkers associated with the GXSTC treatment in the LAD-induced rat CHD model. Finally, the MetaboAnalyst 4.0 platform was used for metabolic pathway enrichment analysis.

**Results**: GXSTC was able to regulate the contents of CK, LDH and AST; restore impaired cardiac function; and significantly reduce the myocardial infarction area in model rats. Twenty-two biomarkers and nine metabolic pathways of GXSTC in the treatment of CHD were identified through UHPLC-MS/MS-based untargeted metabolomics analysis.

**Conclusion**: GXSTC regulates metabolic disorders of endogenous components in LAD-induced CHD rats. The anti-CHD mechanism of GXSTC is mainly related to the regulation of amino acid, lipid and hormonal metabolism. This study provides an overall view of the mechanism underlying the action of GXSTC against CHD.

## Introduction

Coronary heart disease is a common cardiovascular disease, mainly due to coronary atherosclerosis. In the past century, the incidence of CHD has been increasing, and it has become one of the major causes of death in many countries (Yang et al., 2019). Currently, the conventional drugs used to treat CHD are mainly Western drugs, which usually aim to act on individual targets, such as beta-blockers, nitrates, statins, calcium channel blockers or angiotensin-converting enzyme inhibitors. The side effects of these drugs are obvious in the long term. Consequently, researchers have been constantly optimizing strategies for the prevention and treatment of CHD. More recently, traditional Chinese medicine has received great attention because it has a variety of ingredients, can act on multiple targets and pathways and plays an overall regulatory role (Hu et al., 2019). Recent studies have confirmed that compared to Western medicine, TCM has fewer side effects and unique advantages for treating CHD (Zhang et al., 2019; Leung and Xu, 2020). Therefore, the exploration of the pharmacological mechanisms of TCM may provide insights into the development of new drugs.

Guan-Xin-Shu-Tong capsule, a Chinese medicinal formula, has been clinically used to treat CHD for many years. GXSTC is composed of five herbal medicines: Fructus Choerospondiatis (Guangzao), Salvia Miltiorrhiza (Danshen), Flos Caryophylli (Dingxiang), Borneolum (Bingpian) and Concretio Silicea Bambusae (Tianzhuhuang). GXSTC can reduce the levels of interleukin-1β (IL-1β), interleukin-6 (IL-6) and other inflammatory factors in the serum of rats after myocardial infarction and can also prevent apoptosis of myocardial cells (Liang et al., 2012). In addition, GXSTC can significantly decrease the expression level of NADPH oxidase in the myocardium of ischemic rats and play a protective role in the myocardium by exerting antioxidative activity (Cao et al., 2014). However, the systematic mechanism of action, specific active ingredients and *in vivo* targets of GXSTC remain to be explored.

Metabolomics is the profiling of metabolites in biofluids, cells and tissues and is routinely applied as a tool for biomarker discovery. Metabolomics can also be used to understand the system-level effects of metabolites (Johnson et al., 2016; Wishart, 2016; McGarrah et al., 2018). Metabolomics can be divided into untargeted and targeted metabolomics (Cui et al., 2018). The untargeted metabolomics has the advantages of high efficiency, sensitivity and comprehensiveness, which has led it to be increasingly used in revealing the complex action mechanism of TCM (Guijas et al., 2018).

In this study, we used an UHPLC-MS/MS-based untargeted metabolomics to identify the key biomarkers, metabolic pathways and mechanisms of action GXSTC in the treatment of CHD.

## Materials and Methods

### Materials and Reagents

GXSTC was purchased from Shaanxi Buchang Pharmaceutical Co., Ltd (Shaanxi, China; batch number: 20190205). The assay kits for the determination of creatine kinase, aspartate transaminase and lactate dehydrogenase activities were supplied by the Nanjing Jiancheng Bioengineering Institute (Nanjing, China). 2,3,5-Triphenyltetrazolium chloride (TTC) and Evans Blue (EB) were purchased from Sigma Chemical Co. (St Louis, MO, United States). High-performance liquid chromatography (HPLC)-grade methanol, HPLC-grade formic acid and ammonium acetate were supplied by Thermo Fisher Scientific (Waltham, MA, United States). Ultrapure water was generated by a Millipore system (Bedford, MA, United States).

### Experimental Animals

Male Sprague-Dawley rats (200 ± 15g at 6–7weeks) were purchased from SPF Biotechnology Co., Ltd (Beijing, China). All animals were housed under standard laboratory conditions of temperature (25 ± 2°C), constant humidity (55 ± 5%) and light (12h dark/light cycle) with freely available food and purified water. After adaptive feeding for one week, rats were randomly selected and subjected to sham surgery or CHD. The CHD rat model was established by ligation of the left anterior descending coronary artery. Briefly, a left thoracotomy was performed after rats were anesthetized with 2% isoflurane. The left anterior descending coronary artery was then ligated with a 5–0 polypropylene suture. After the ligation, the heart was placed back in the chest cavity, the air that entered the chest cavity was expelled, the chest cavity was closed, and the skin was sutured. For the sham-operated group, rat LADs were only threaded and not ligated, and the remaining procedures were the same as those in the CHD group. After the operation, electrocardiogram monitoring was performed. T wave changes and ST segment elevation in the rats in the CHD group indicated that the model was successfully constructed; otherwise, it was rejected (Chen et al., 2017).

The CHD-operated rats were divided into the CHD model group (Model, *n* = 6) and the GXSTC administration group (Treat, *n* = 6) according to a random number control table. Rats without ligation were placed in the sham-operated group (Sham, *n* = 6). The rats in the Treat group received a dose of 1.2 g/kg/d GXSTC at a drug concentration of 0.06 g/ml, which was continuously administered for four weeks. The rats in the Sham and Model groups were given the same volume of normal saline via intragastric administration.

All experimental rats were handled humanely, and all procedures were strictly performed according to the ARRIVE guidelines and approved by the Animal Ethics Committee of the Institute of Radiation Medicine, Chinese Academy of Medical Sciences (Approval No. IRM-DWLL-2019075, Tianjin, China).

### Index Detection

#### Echocardiography Detection

After two weeks, each rat was subjected to echocardiography using a Visual Sonics ultrahigh-resolution small animal ultrasound imaging system (VisualSonics, Toronto, Canada), and the left ventricular ejection fraction (EF) and left ventricular fractional shortening (FS) were two indicators used to evaluate the heart function of rats (Sciarretta et al., 2018). After acquiring a myocardial M-shaped sample line, ultrasonic index measurements and calculations were performed, including left ventricular end diastolic diameter (LVEDd), left ventricular end systolic diameter (LVESd), left ventricular end diastolic volume (LVEDv), left ventricular end systolic volume (LVESv), EF = [(LVEDv−LVESv)/LVEDv]×100% and FS = [(LVEDd−LVESd)/LVEDd]×100%.

#### Biochemical Index Detection

One milliliter of blood from each rat s ocular vein was placed into a 2 ml centrifuge tube and allowed to stand at room temperature for 30 min to coagulate and layer. Then, the samples were centrifuged at 3,000 rpm for 15 min at room temperature, and the upper serum was drawn into a 1.5 ml centrifuge tube. The contents of CK, AST and LDH in each rat serum sample were determined according to the instructions of the kit. The serum used to determine the LDH content was diluted 40 times with normal saline.

#### Myocardial Infarct Size Detection

To more intuitively evaluate the therapeutic effect of GXSTC on CHD rats, we produced myocardial infarct slices from rat hearts and measured the area of myocardial infarct in each group by EB and TTC double staining (Zhu et al., 2015). Rats were injected with 2.5 ml EB at a concentration of 2% through the tail vein. Rats were sacrificed after 30 min of circulation, and the heart was quickly removed. After freezing at −20°C for 30 min, the heart was cut into five pieces on the vertical longitudinal center axis. Then, a 1% TTC solution was added, and the pieces were wrapped in foil to protect them from light. All the pieces were placed in a water bath and incubated at 37°C for 15 min. The pieces were then fixed for 12 h, and then, a camera was used to take pictures, keeping the distance between the lens and each slice unchanged at ×5 magnification. Image-Pro Plus 6.0 image processing software was used to process the slice photos. The area stained by EB in the slice was marked as the area not at risk (ANAR), and the rest of the slice was marked as the area at risk (AAR). The AAR was composed of an off-white area (infarct area) and a red area (ischemic but not infarct area). Myocardial infarct size = infarct area/ischemic area × 100%.

### Plasma Samples Preparation for Metabolic Profiling

Plasma samples were removed and placed at 4°C to slowly thaw. One hundred microliters was aspirated and placed in a 1.5 ml centrifuge tube, and 400 μL of MS grade methanol was added and vortexed for 30 s. After being left to stand in an ice bath for 5 min, the tube was centrifuged at 15,000 rpm at 4°C for 10 min in a low-temperature/high-speed centrifuge. The supernatant was mixed with 53% MS-grade methanol. The tube was centrifuged again at 15,000 rpm for 10 min at 4°C in the low-temperature high-speed centrifuge, and the supernatant was collected for subsequent UHPLC-MS analysis.

The quality control (QC) samples were prepared by mixing equal volumes (10 μL) of all the plasma samples. Five QC samples were injected before the test sample to monitor the status of the instrument and balance the chromatography-mass spectrometry system. During the testing of the test samples, four QC samples were randomly injected to evaluate the stability of the chromatography-mass spectrometry system and the reliability of the experimental data throughout the experiment.

### UHPLC-MS/MS Conditions

Chromatographic separation of all rat plasma samples was completed using a Thermo Vanquish Flex Ultra Performance Liquid Chromatograph (Thermo Fisher) and Thermo Hyperil Gold column (100 mm × 2.1 mm×1.9 µm). The column temperature was 40°C, and the flow rate was 0.2 ml/min. Eluent A was 0.1% formic acid, and eluent B was methanol. The solvent gradient was set as follows: 0–1.5 min, 2% B; 1.5–12.0 min, 2–100% B; 12.0–14.0 min, 100% B; 14.0–14.1 min 100–2% B; and 14.1–17 min 2% B.

Rat plasma samples were separated by UHPLC and analyzed by a Thermo Scientific quadrupole Orbitrap mass spectrometer. The electrospray ion source (ESI) positive ion (pos) and negative ion (neg) modes were used for detection, and the scanning range was m/z 70–1,050, spray voltage was 3.2 kV, sheath gas flow rate was 35 arb, aux gas flow rate was 10 arb, and capillary temperature was 320°C.

### Data Analysis

First, Compound Discoverer 3.0 (CD) software was used to preprocess the obtained mass spectrometry data. The specific operations were as follows: the peak alignment standard was a retention time deviation of 0.2 min and mass deviation of 5 ppm. At the same time, peak extraction was performed according to the requirements of a mass deviation of 5 ppm, signal intensity deviation of 30%, signal-to-noise ratio of 3, and minimum signal intensity of 100,000. Then, the peak area was quantified and normalized.

Natural separation of all the tested groups was performed by the PCA model. The value of variable importance in the projection (VIP) from the PLS-DA model, the ratio of the mean of all the biological replicate quantitative values in the comparison group of each metabolite (fold change, FC), and the *p* value for univariate analysis (*t*-test) were used as parameters to screen different metabolites from different sample groups. Metabolites with VIP >1 and FC > 1.2 or FC < 0.833 and *p* < 0.05 were considered to be potential biomarkers. These potential biomarkers were then annotated and subjected to pathway analysis performed through the online databases mzCloud (https://www.mzcloud.org/), SMPDB (https://smpdb.ca/), HMDB (http://www.hmdb.ca/), and KEGG (http://www.genome.jp/kegg/).

### Statistical Analysis

All experimental data are expressed as the mean ± standard deviation (mean ± SD). GraphPad Prism 5.0 (La Jolla, CA) statistical analysis software was used to perform t-tests and one-way ANOVA. *p* < 0.05 indicates statistical significance.

## Results

### Echocardiography Detection

**FIGURE 1:**
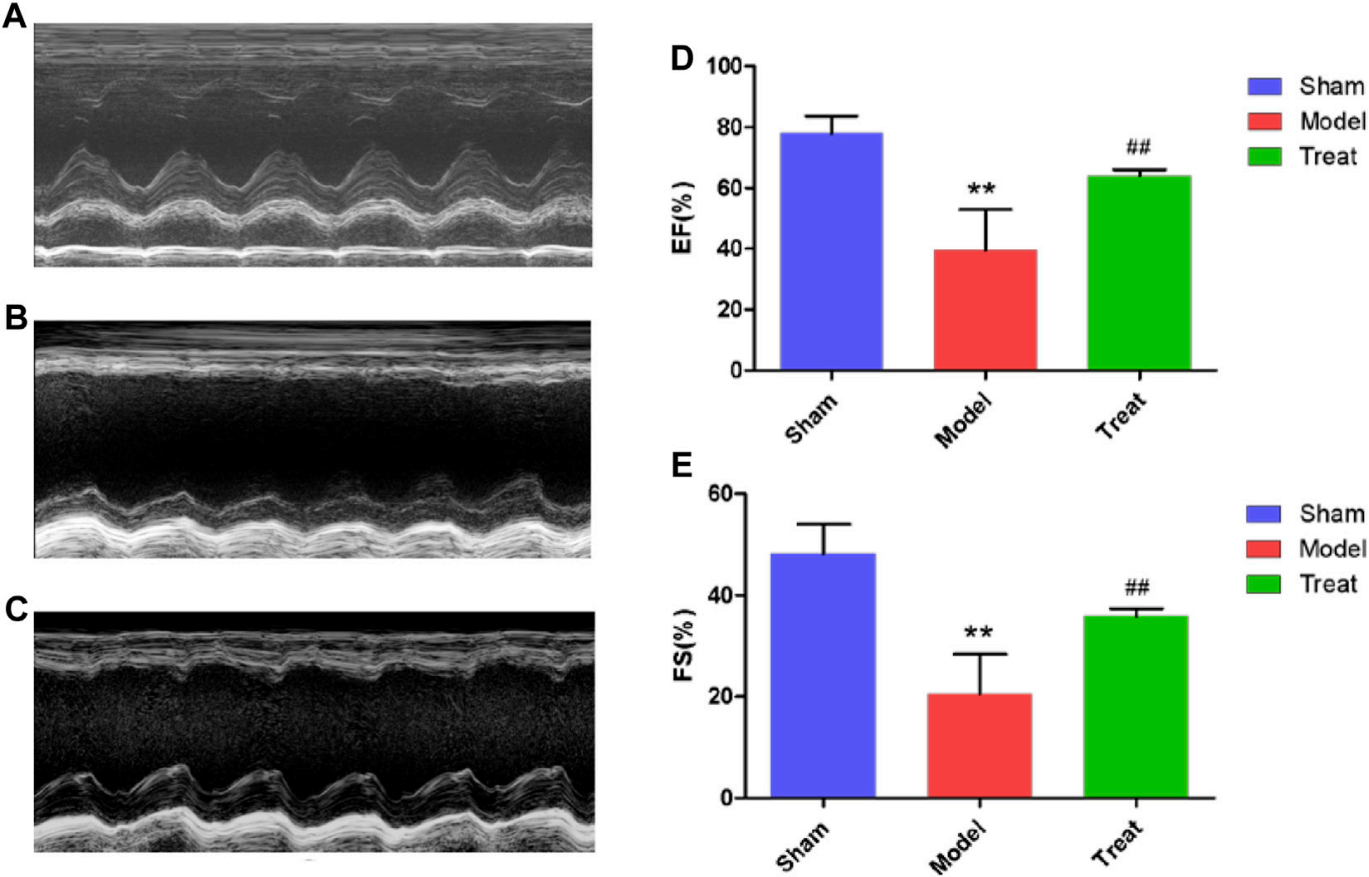
**(A,B and C)** Representative images of echocardiography **(D and E)** EF and FS were measured by echocardiography. Data were expressed as mean ± SD (*n* = 6), **p < 0.01 compared with the Sham group, ##*p* < 0.01 compared with the Model group.

To evaluate the improved effect of GXSTC on cardiac function in the model rats with CHD, echocardiography was performed in all rats after two weeks of administration. As shown in Figure 1, the echocardiography waveform of the sham group rats was clear and regular, and the EF and FS values were 77.96% ± 2.540 and 48.29% ± 2.544, respectively. All the data indicated that the systolic and pumping functions of the rats were good. Compared with the Sham group, the echocardiography waveform of the Model group was significantly changed, with EF and FS values decreasing to 39.51% ± 5.960 and 20.54% ± 3.513, respectively, indicating that the cardiac function of the Model group had been seriously damaged and that the CHD Model was successfully constructed. Compared with the Model group, the echocardiography waveform of the Treatment group was improved, and the EF and FS values increased to 63.94% ± 0.9126 and 35.84% ± 0.6803, respectively, which indicated that GXSTC improved cardiac function in rats with CHD.

### Biochemical Index Detection

**FIGURE 2:**
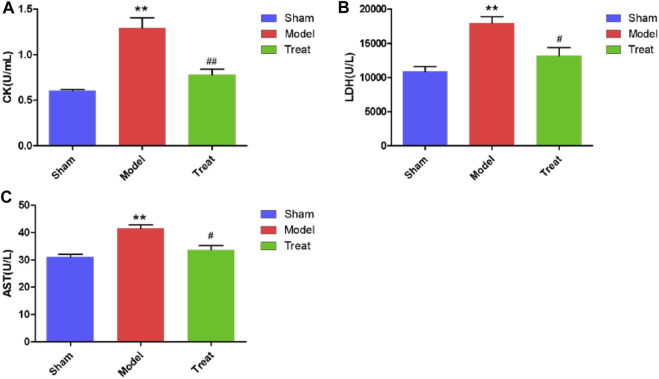
CK, LDH and AST levels in Sham group, Model group and Treat group. Data were expressed as mean ± SD (*n* = 6), *p < 0.05, **p < 0.01 compared with the Sham group, #*p* < 0.05, ##*p* < 0.01 compared with the Model group.

The elevated levels of CK, LDH and AST in serum samples of rats are important markers after myocardial infarction because severe ischemia and hypoxia of the myocardium can cause myocardial cell necrosis, which causes CK, LDH and AST to be released from the myocardium into the blood circulation system. As shown in Figure 2, compared with the Sham group, the serum levels of CK, LDH and AST were significantly increased in the Model group. These results indicated that the CHD model in rats after LAD was successfully constructed, but the level of autonomous recovery without drug intervention was poor. Additionally, in the Treatment group, the CK, LDH and AST levels were significantly decreased, which indicated that GXSTC treatment significantly reduced these values compared to those in the Model group, which implied that GXSTC had a positive therapeutic effect on rats with CHD.

### Myocardial Infarct Size Detection

**FIGURE 3:**
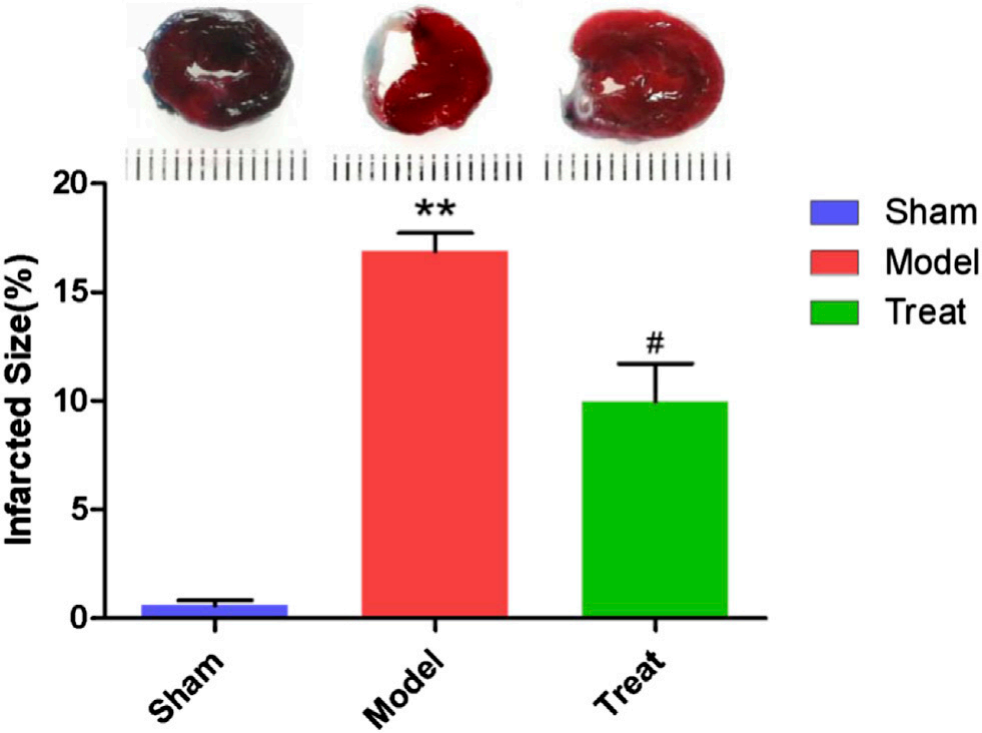
Representative images of EB/TTC double staining of rat heart sections and myocardial infarction area of each group. Data were expressed as mean ± SD (*n* = 6), **p < 0.01 compared with the Sham group, #*p* < 0.05 compared with the Model group.

In this study, EB/TTC double staining was used to observe myocardial infarction of rats in different groups, and the results are shown in Figure 3. In the Sham group, no obvious infarctions were observed, and more than half of the area was stained by EB as blue-purple, indicating that no obvious ischemia occurred in the hearts of the Sham group rats, and the hearts remained relatively complete in shape and function. The entire myocardium of the Model group was stained brick red by TTC, indicating that the heart was basically in a state of ischemia. Moreover, significant collapse and atrophy of the left ventricle of rats could be observed, and the myocardial infarct area reached 16.90% ± 0.83. Compared with the Model group, the area of myocardial infarction in the Treatment group was significantly reduced (9.97% ± 1.73).

### Metabolomic Study of Plasma Samples

#### Method Validation

To ensure the stability of the chromatography-mass spectrometry system and the reliability of the experimental data during the experiment, four QC samples were randomly injected during the testing of the plasma samples, and the total ion current diagram was obtained. The response intensity and retention time of each chromatographic peak of the four QC samples were basically the same, indicating that the stability of the chromatography-mass spectrometry system was good throughout the experiment. Then, the peak area was used to calculate the Pearson correlation coefficient among the four QC samples. The results showed that the *R* ^2^ between each sample was greater than 0.985, indicating that the QC samples were highly correlated and the metabolome results were repeatable and stable.

#### Multivariate Statistical Analysis

**FIGURE 4:**
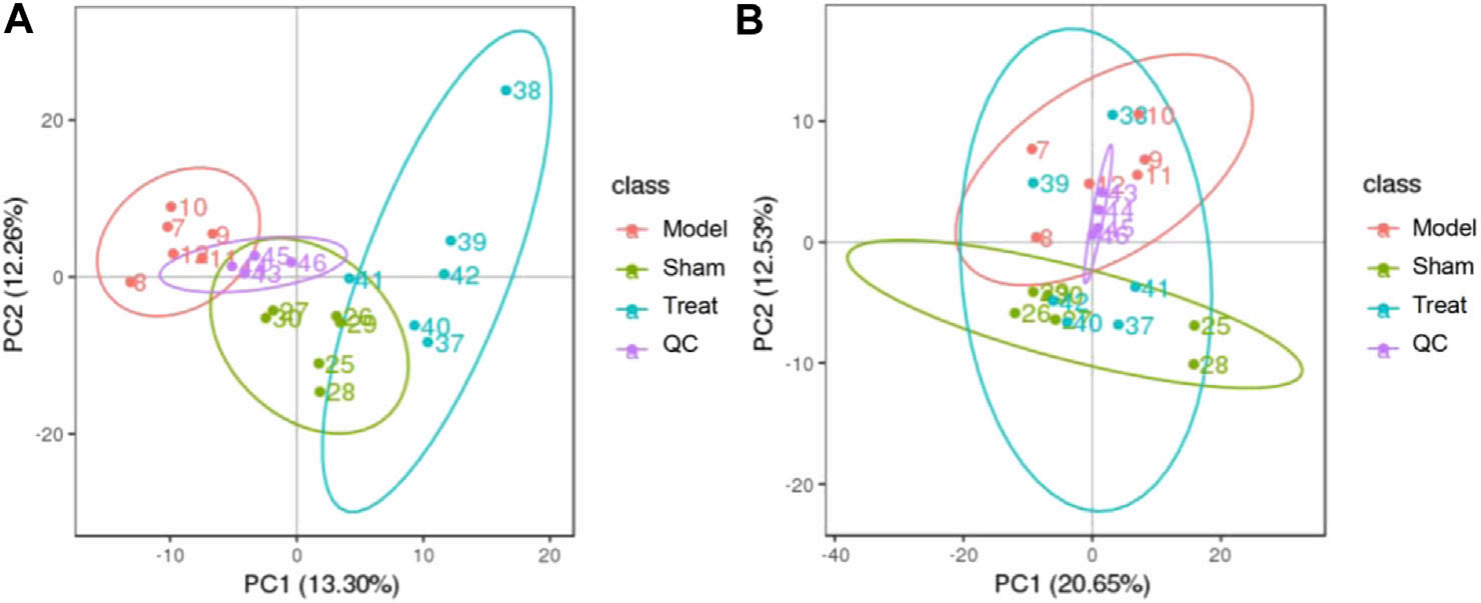
PCA score plots of the plasma samples from Sham group, Model group, Treat group, QC samples in positive mode **(A)** and negative modes **(B)**. The ellipse represents the confidence interval of 95%.

PCA is an unsupervized data analysis method that can generally reflect metabolic differences among samples of different groups and the degree of variation among samples of each group. In this work, PCA models of the plasma metabolomic data were constructed to determine the natural separation of metabolites in different groups. As shown in Figure 4, the metabolic profile of the rats in the Model group deviated from that of the rats in the Sham group, suggesting that significant biochemical changes were induced by CHD. Moreover, the mean center of spots in the Treatment group was fairly different from that in the Model group and was close to that in the Sham group, indicating that GXSTC treatment of CHD rats had a positive effect on metabolites. In addition, QC samples were concentrated in the middle area of the tested samples, which indicated that the QC samples had good repeatability and stability.

**FIGURE 5:**
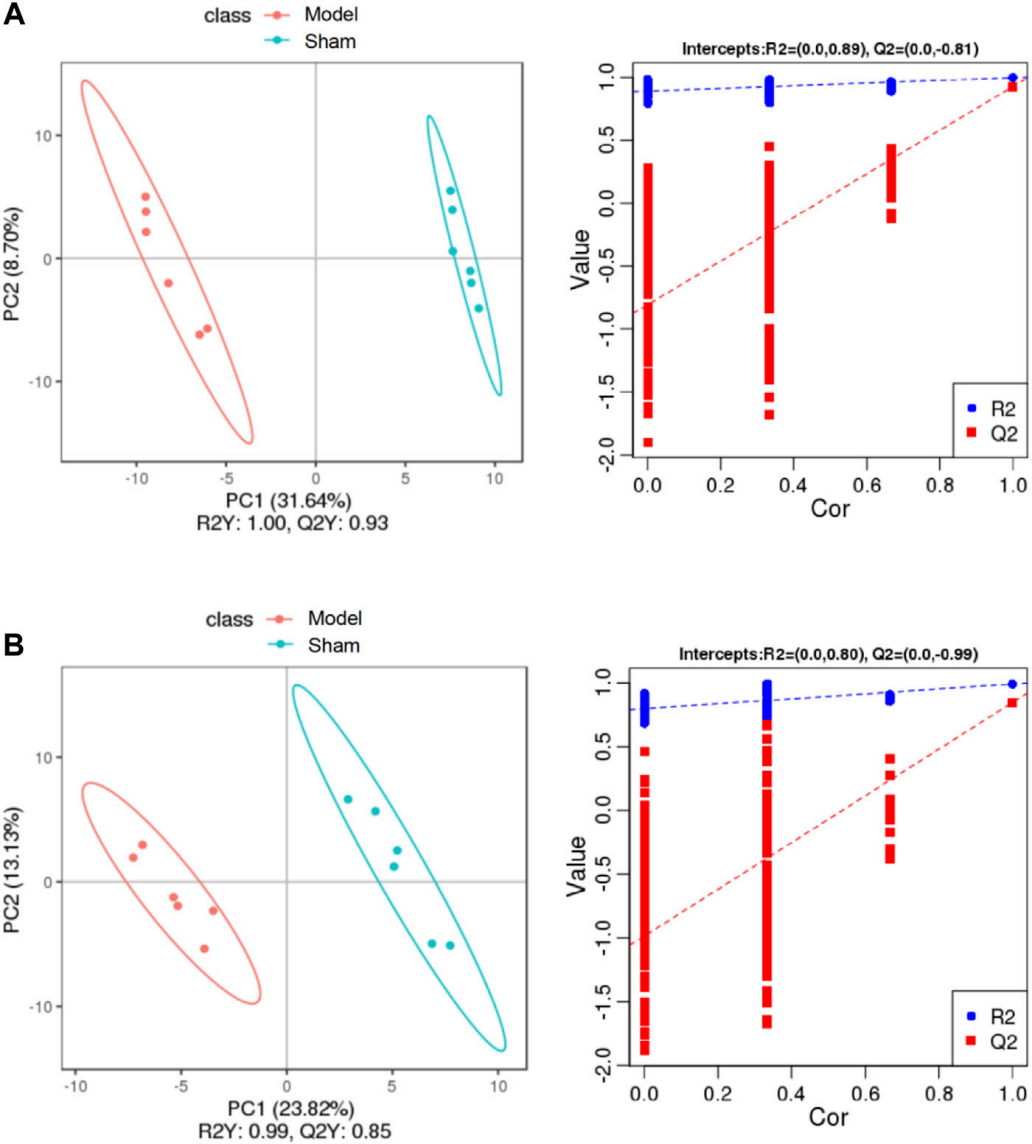
PLS-DA score plots of the plasma samples from Sham group, Model group in positive mode **(A)** and negative mode **(B)**. The corresponding validation plots based on 200 times permutation tests of the PLS-DA models in positive mode **(A)** and negative mode **(B)**. The ellipse represents the confidence interval of 95%.

To discriminate the differential metabolites between each pair of groups, supervised PLS-DA analyses were performed on the Model vs. Sham and Model vs. Treatment groups (Figures 5A,B; Supplementary Figure S1C,D). At the same time, a 7-fold cross-validation was conducted to obtain the model evaluation parameters *R* ^2^ and Q^2^ to test the reliability of the PLS-DA model. The parameters were *R* ^2^ = 1.00 and Q^2^ = 0.93 between the Sham and Model groups in the positive ion mode, *R* ^2^ = 0.99 and Q^2^ = 0.85 in the negative ion mode, *R* ^2^ = 1.00 and Q^2^ = 0.94 between the Treatment and Model in the positive ion mode, and *R* ^2^ = 0.99 and Q^2^ = 0.84 in the negative ion mode, which revealed the good stability and high predictability of the results. Then, the potential overfitting of the PLS-DA model was assessed by two hundred permutation tests, in which all the *R* ^2^ and Q^2^ values were lower than the original values, indicating the reliability of the established discriminant model.

### Screening and Identification of Differential Metabolites

**FIGURE 6:**
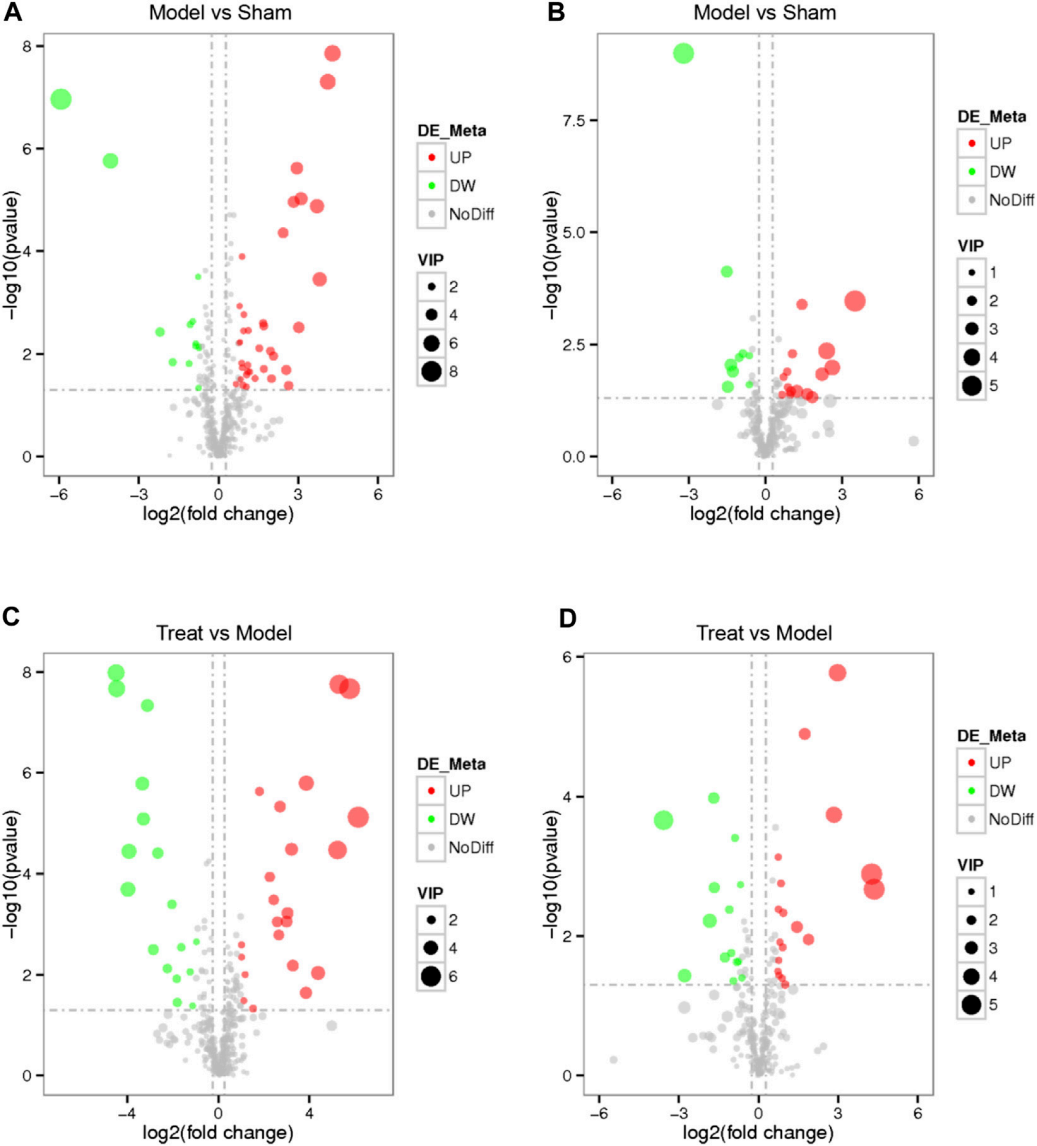
Volcano map of every differential metabolite among the Model VS. Sham in positive mode **(A)**, Model VS. Sham in negative mode **(B)**, Treat VS. Model in positive mode **(C)** and Treat VS. Model in negative mode **(D)**. Each dot represents a metabolite, red represents a significantly up-regulated metabolite, green represents a significantly down-regulated metabolite, the size represents the VIP.

Based on the VIP value of the first principal component of the PLS-DA model, FC value and *p* value, a significant difference in the metabolites of different groups was identified. Using VIP >1.0, FC > 1.2 or FC < 0.833 and *p* < 0.05 as the threshold, 73 differential metabolites were obtained (49 from the positive mode and 24 from the negative mode) between the Model and Sham groups. Likewise, 71 differential metabolites conforming to parameter thresholds were obtained (39 from the positive mode and 32 from the negative mode) between the Treatment and Model groups. To show the overall distribution of the differential metabolites more intuitively, a volcano map was constructed. As shown in Figure 6, each dot represents a metabolite, with those in red representing a significantly upregulated metabolite due to VIP >1.0, *p* < 0.05 and FC > 1.2 and those in green representing a significantly downregulated metabolite due to VIP >1.0, *p* < 0.05 and FC < 0.833.

**FIGURE 7:**
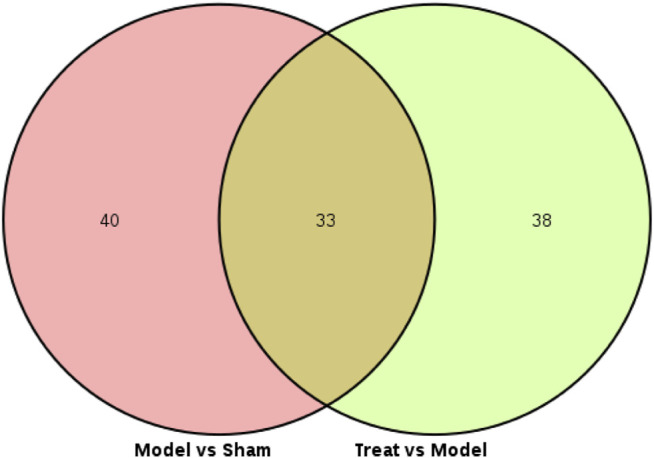
Venn diagram of every differential metabolites between the Model VS. Sham and Treat VS. Model.

**FIGURE 8:**
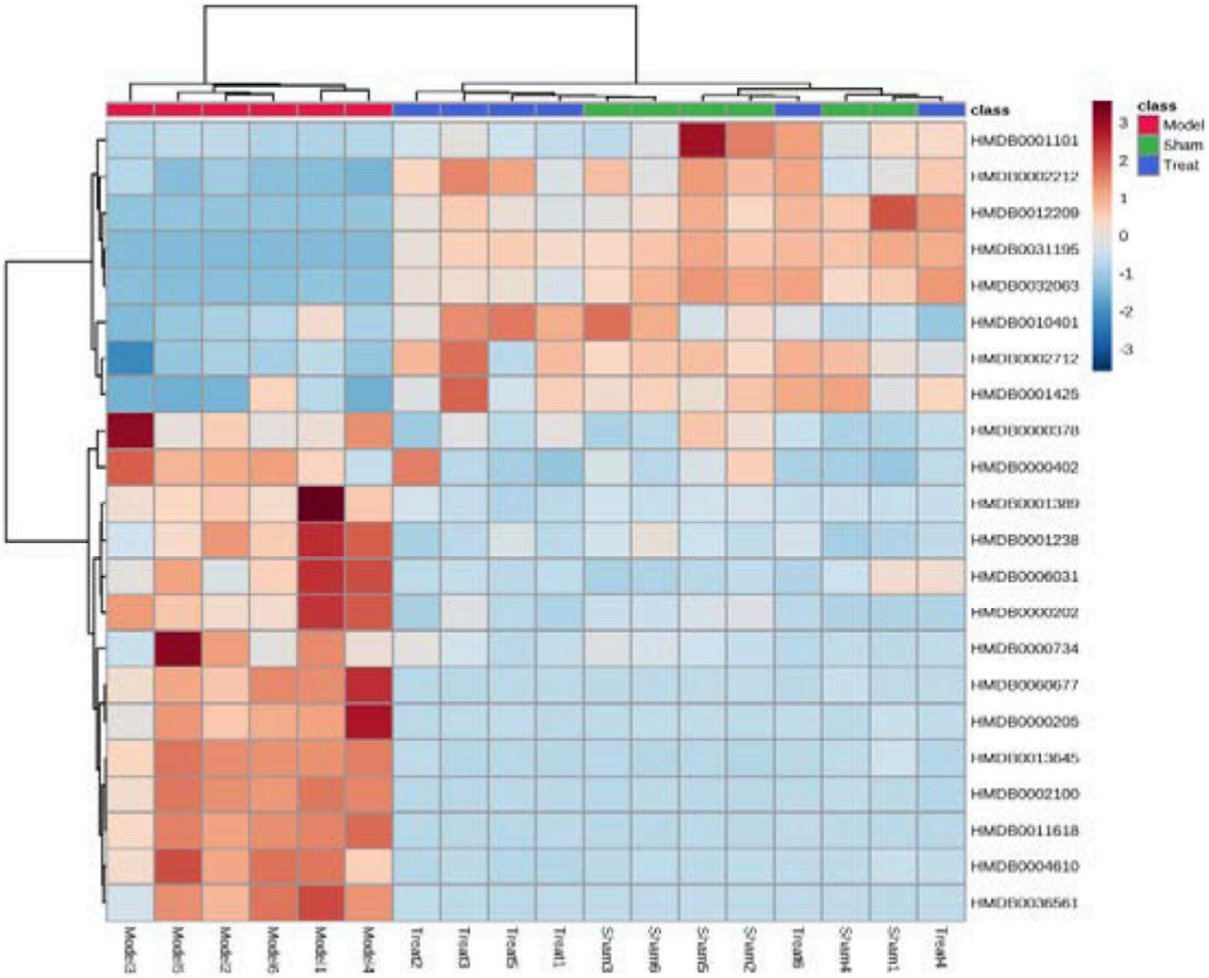
Hierarchical cluster analysis heat map of potential biomarkers in each group. Horizontal is the clustering of potential biomarkers, vertical is the clustering of different groups of samples, red represents significantly up-regulated biomarkers, blue represents significantly down-regulated biomarkers, and the biomarkers clustered in the same cluster have similar expression patterns.

Subsequently, a Venn diagram was used to integrate overlapping metabolites of each pairwise comparison. The result is shown in Figure 7. A total of 33 overlapping differential metabolites were obtained between the two compared groups. These 33 differential metabolites were preliminarily annotated using the KEGG, HMDB and METLIN databases. Some of the identified metabolites were further confirmed using authentic reference material. Finally, 22 biomarkers were identified as potential biomarkers associated with CHD, including 13 in the positive mode and nine in the negative ion mode. Detailed information of the biomarkers is provided in Table 1. The results of the biomarkers showed that all 22 biomarkers in plasma samples of the Treatment group had a significant callback compared with the Model group. Furthermore, according to the abundance value of every potential biomarker, a heat map was constructed to observe the relationship among the groups directly. As depicted in Figure 8, these potential biomarkers showed distinct segregation between the Treatment and Model groups. The content of the potential biomarkers in the Treatment group was closer to that of the potential biomarkers in the Sham group than to that of the potential biomarkers in the Model group.

**TABLE 1:** Potentiale biomarkers associated with GXSTC treatment in rat plasma in positive mode and negative mode.

Mode	Metabolites	HMDB ID	Formula	tR/min	Mass (m/z)	FC_S_	FC_T_	Model vs sham	Treat vs model
pos	Palmitoylethanolamide	HMDB0002100	C_18_H_37_NO_2_	11.88	299.28	17.22	0.05	Up^b^	Down^d^
	11-Ketoetiocholanolone	HMDB0006031	C_19_H_28_O_3_	13.91	286.19	3.25	0.32	up^b^	down^d^
	Melatonin	HMDB0001389	C_13_H_16_N_2_O_2_	8.67	232.12	3.20	0.24	up^b^	down^d^
	Isophorone	HMDB0031195	C_9_H_14_O	10.67	138.10	0.02	54.56	down^b^	up^d^
	2,6-Dimethylaniline	HMDB0060677	C_8_H_11_N	5.77	121.09	5.38	0.16	up^b^	down^d^
	Indoleacrylic acid	HMDB0000734	C_11_H_9_NO_2_	11.05	187.06	3.25	0.28	Up^a^	Down^c^
	p-Anisic acid	HMDB0001101	C_8_H_8_O_3_	9.00	152.05	0.30	2.26	down^a^	Up^d^
	2-Methylbutyroylcarnitine	HMDB0000378	C_12_H_23_NO_4_	9.95	245.16	2.13	0.42	up^a^	down^d^
	All-trans-13,14-dihydroretinol	HMDB0011618	C_20_H_32_O	11.09	288.24	19.50	0.04	up^b^	down^d^
	Phenylpyruvic acid	HMDB0000205	C_9_H_8_O_3_	12.92	164.05	13.01	0.07	up^b^	down^d^
	N,N-Dimethylsphingosine	HMDB0013645	C_20_H_41_NO_2_	13.16	327.31	7.70	0.11	up^b^	down^d^
	Diethylphosphate	HMDB0012209	C_4_H_11_O_4_P	9.99	154.04	0.06	14.55	down^b^	up^d^
	Phytosphingosine	HMDB0004610	C_18_H_39_NO_3_	12.73	317.29	7.08	0.10	up^b^	down^d^
neg	Polygalitol	HMDB0002712	C_6_H_12_O_5_	1.41	224.09	0.54	1.90	down^b^	up^d^
	Methylmalonic acid	HMDB0000202	C_4_H_6_O_4_	1.24	118.03	2.68	0.31	up^b^	down^d^
	4-tert-Butylphenol	HMDB0032063	C_10_H_14_O	12.32	150.10	0.11	7.86	down^b^	up^d^
	Arachidic acid	HMDB0002212	C_20_H_40_O_2_	14.49	312.30	0.35	3.34	down^b^	up^d^
	Lysophosphatidylcholine (22:4)	HMDB0010401	C_30_H_54_NO_7_P	14.96	631.39	0.64	1.66	down^a^	up^c^
	N-Acetylserotonin	HMDB0001238	C_12_H_14_N_2_O_2_	12.42	218.11	2.07	0.46	up^b^	down^d^
	Estrone sulfate	HMDB0001425	C_18_H_22_O_5_S	1.45	350.12	0.39	2.72	down^b^	up^d^
	2-Isopropylmalic acid	HMDB0000402	C_7_H_12_O_5_	1.27	1.80	0.56	176.07	up^a^	down^c^
	Agnuside	HMDB0036561	C_22_H_26_O_11_	11.55	466.15	11.32	0.08	up^b^	down^d^

Note, the FC_s_ and FC_T_ values represent the fold change of the Model group compared to the Sham group or Treat group, respectively.

^a^ *p* < 0.05.

^b^ *p* < 0.01 compared with the sham group.

^c^ *p* < 0.05.

^d^ *p* < 0.01 compared with the model group.

### Metabolic Pathway Analysis of Potential Biomarkers

**FIGURE 9:**
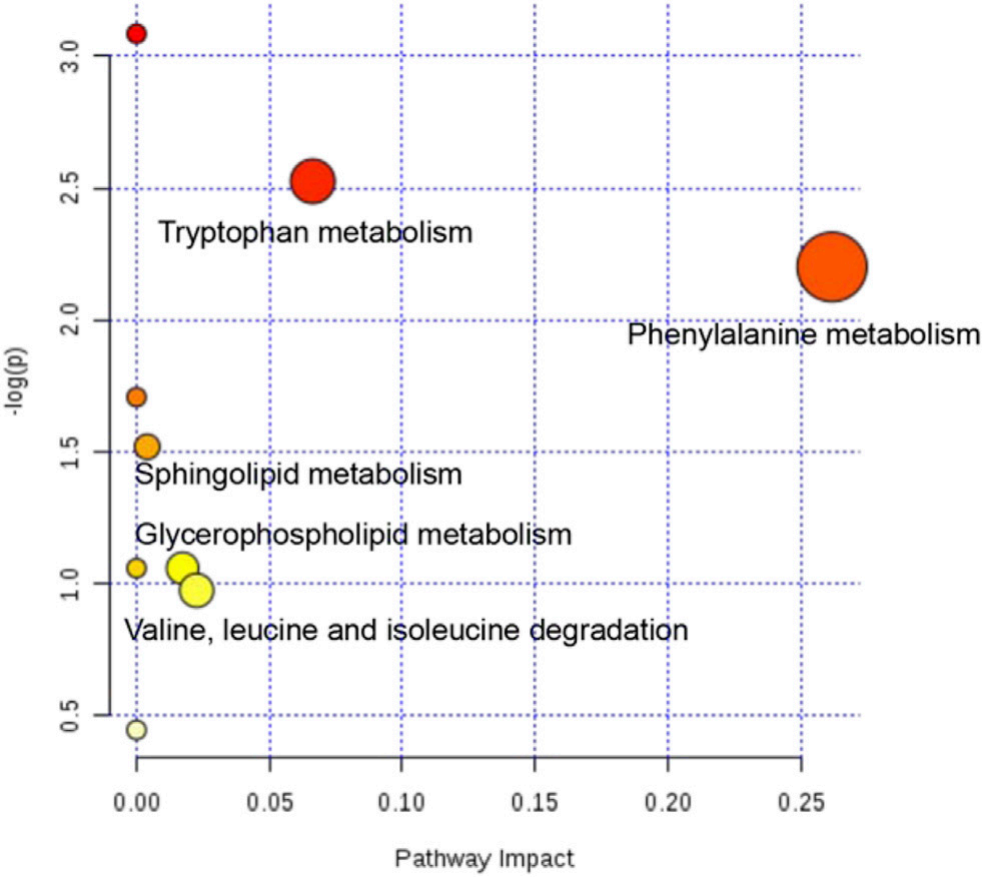
Analysis diagram of metabolic pathway of potential biomarkers. Each point represents one metabolic pathway. The size of dot and the shade of color are positively related to the effect on metabolic pathway.

The metabolic pathways of 22 potential biomarkers were analyzed by MetaboAnalyst (https://www.metaboanalyst.ca/) to evaluate the importance of the pathways in the efficacy of CHD. As shown in Figure 9, these biomarkers were distributed in various metabolic pathways of the body, including phenylalanine metabolism, tryptophan metabolism, valine, leucine and isoleucine degradation, glycerophospholipid metabolism, sphingolipid metabolism, phenylalanine, tyrosine and tryptophan biosynthesis, retinol metabolism, biosynthesis of unsaturated fatty acids, and steroid hormone biosynthesis. These pathways might denote their potential as the targeted pathways of GXSTC against CHD.

## Discussion

TCM, which is characterized by multicomponent, multitarget, overall regulation and lower adverse reactions than Western medicine, has unique advantages for treating CHD, which requires long-term treatment. However, the composition of TCM is so complicated that it is too difficult and inefficient to explore its molecular mechanisms through conventional experimental methods. Metabolomics has holistic and systematic characteristics, which are highly consistent with the concept of TCM regulating the overall balance of the body. Therefore, we used a plasma metabolomics method to reveal the overall treatment mechanism of action GXSTC in CHD rats.

LAD-induced CHD rats exhibited obvious ST-segment elevation along with myocardial damage and hypofunction, potentially making them a useful model of human CHD. In the present study, decreased EF and FS, as well as increased myocardial infarct size and enzyme activities of serum CK, LDH and AST were observed in LAD-induced model rats. After treatment with GXSTC, EF and FS in the Treatment group were markedly increased. The area of myocardial infarction and enzyme activities of serum CK, LDH and AST in the Treat group were decreased compared with those of the Model group. These results are consistent with previous reports (Liu et al., 2014; Yang et al., 2017; Yang et al., 2018), indicating that GXSTC has a significant therapeutic effect on CHD.

To deeply explore the therapeutic effect of GXSTC in CHD rats, a UHPLC-MS/MS-based plasma metabolomics method combined with a multivariate statistical analysis was established. With the metabolomics approach, the deviations caused by CHD were significantly ameliorated after treatment with GXSTC, suggesting that its cardioprotective effects are most likely to ameliorate the metabolic disorders induced by LAD. Twenty-two potential biomarkers were identified as being related to GXSTC against CHD, and they were involved in nine metabolic pathways. These biomarkers were involved in a variety of physiological and biochemical processes, especially lipid metabolism, amino acid metabolism and hormone metabolism.

Lipid metabolism disorder is an independent risk factor for CHD and the pathological basis of coronary atherosclerosis (Ference et al., 2019). Ameliorating lipid metabolism disorder and keeping blood lipids at the appropriate level can effectively prevent and treat CHD (Lee et al., 2017). Changes in sphingolipid and glycerolipid metabolism can well reflect lipid metabolism disorders in the body under pathological conditions (Harshfield et al., 2019). In our study, compared with the Sham group, the phytosphingosine content was increased and lysophosphatidylcholine (22:4) was significantly reduced in the Model group, while both metabolites returned the normal levels in the Treat group, suggesting that GXSTC has a regulatory effect on lipid metabolism disorders.

As important enzyme substrates and regulators, amino acids play important roles in many metabolic pathways, which contain important biochemical information reflecting the metabolic and functional state of the body and play important roles in the normal operation of the immune system and other organs (White and Newgard, 2019). Studies have shown that phenylalanine metabolic disorder is related to the aggravation of oxidative stress reactions, such as lipid peroxidation in the body (Dong et al., 2019). The oxidative stress reaction causes vascular endothelial oxidative damage and then leads to vascular endothelial dysfunction, which is an indicative response in the early stage of many cardiovascular diseases (Zhang et al., 2020). The increased phenylalanine content in the Model group indicated that CHD resulted in abnormal phenylalanine metabolism in the body, while the Treat group raised the phenylalanine content to the normal level and inhibited abnormal phenylalanine metabolism. This result indicated that GXSTC played a role in the regulation of phenylalanine metabolism. In our study, we found that N-acetylserotonin, indoleac acid and melatonin were correlated with tryptophan metabolism. Compared with those in the Sham group, the levels of the metabolites N-acetylserotonin, indoleacid and melatonin in the Model group were elevated, indicating that CHD leads to abnormal tryptophan metabolism in the body, which was consistent with previous experimental results (Liu et al., 2017). All three metabolites in the Treatment group showed a callback trend, indicating that GXSTC could inhibit abnormal tryptophan metabolism and reduce the damage of CHD to the body.

Interestingly, our study also found that GXSTC had a specific effect on ameliorating CHD in the regulation of hormone levels. For example, GXSTC has a certain effect on estrone sulfate levels. Estrone sulfate is a form of estrogen. Studies have shown that estrogen may affect the coagulation system, vascular endothelial function and other systems, directly or indirectly enhancing protective effects on the heart (Dai et al., 2015). A recent prospective study of 2,834 subjects followed for an average of 12.1 years found that higher estrogen levels were associated with a lower risk of CHD (Subramanya et al., 2019). Our study results showed that the level of estrone sulfate in the body was increased after treatment with GXSTC, indicating that GXSTC played a certain role in the regulation of estrogen metabolism. In addition, we also found that the level of 11-ketoetiocholanolone, an endogenous anabolic androgenic steroid, increased after GXSTC treatment. Studies have shown that lower levels of serum endogenous testosterone are associated with a higher risk of diseases such as atherosclerosis, myocardial infarction, chronic heart failure and obesity (Fink et al., 2018; Lorigo et al., 2020), and our results indicated that GXSTC also played a role in the regulation of androgen metabolism.

In summary, for the first time, this study revealed the mechanism by which GXSTC acts on CHD at a holistic metabolic level. We can assume that GXSTC has a unique advantage in the treatment of CHD, and its therapeutic mechanism may be the regulation of amino acid metabolism, lipid metabolism and hormone levels. Our study provides a certain theoretical basis for subsequent research.

## Conclusion

In the present study, biochemical analysis of serum, echocardiography and myocardial tissue sections revealed a significant therapeutic effect of GXSTC against CHD in rats. Subsequently, 22 biomarkers and nine disturbed metabolic pathways in the body during the process of CHD were obtained by UHPLC-MS/MS-based untargeted metabolomics. The analysis results showed that GXSTC could call back the disordered metabolic pathways and thus played a therapeutic role in CHD. We also found that these metabolic pathways were related to amino acid metabolism, lipid metabolism and hormone levels in the body, which fully indicated the overall role of GXSTC in the prevention and treatment of CHD through multiple pathways, multiple levels and multiple targets. To the best of our knowledge, our study is the first to use a metabolomics approach to explain the drug effects of GXSTC on CHD rats. Our results can provide a direction for follow-up studies on the mechanism of GXSTC in the treatment of CHD, which is conducive to the clinical promotion and application of GXSTC.

## Data Availability

The original contributions presented in the study are included in the article/Supplementary Material, further inquiries can be directed to the corresponding authors.
