# Supplementary material for: Study of the Mechanism of Action of Guanxin Shutong Capsules in the Treatment of Coronary Heart Disease Based on Metabolomics
Source: Front Pharmacol. 2021 Mar 25;12:650438. doi: 10.3389/fphar.2021.650438 (PMC8048374; doi:10.3389/fphar.2021.650438)
Supplement: Supplementary file 1 [file image1.pdf]

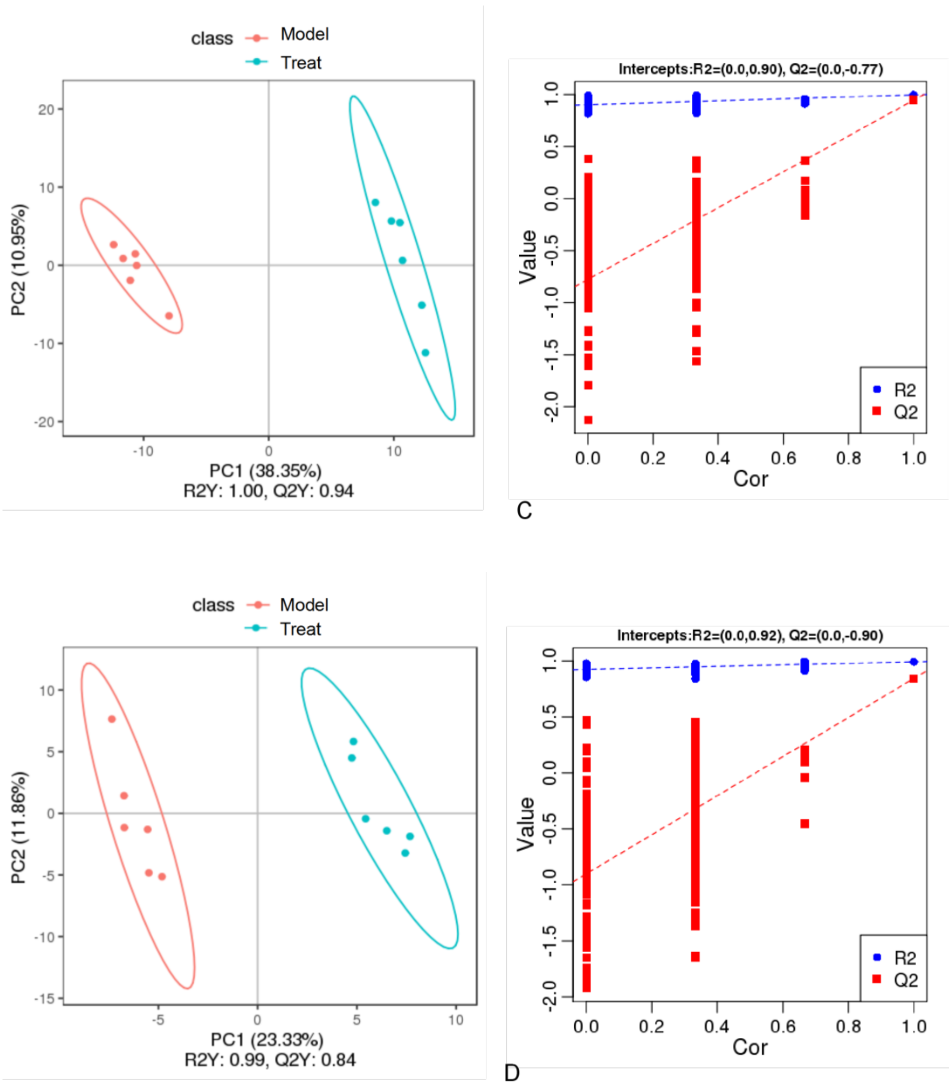

FIGURE S1 PLS-DA score plots of the plasma samples from Model group, Treat group in positive mode (C) and negative mode (D). The corresponding validation plots based on 200 times permutation tests of the PLS-DA models in positive mode (C) and negative mode (D). The ellipse represents the confidence interval of 95%.
